# Intermittent and temporally variable bioturbation by some terrestrial invertebrates: implications for ichnology

**DOI:** 10.1007/s00114-023-01833-0

**Published:** 2023-03-07

**Authors:** Shannon Hsieh, Weronika Łaska, Alfred Uchman

**Affiliations:** 1 Faculty of Geography and Geology, Institute of Geological Sciences, Jagiellonian University, Gronostajowa 3a, 30-387 Kraków, Poland; 2 Institute of Evolutionary Biology, Faculty of Biology, Biological and Chemical Research Centre, University of Warsaw, Warsaw 101, 02-089 Żwirki i Wigury, Poland

**Keywords:** Beetle, Bioturbation, Ichnology, Movement ecology, Terrestrial invertebrates

## Abstract

Bedding planes and vertical sections of many sedimentary rock formations reveal bioturbation structures, including burrows, produced by diverse animal taxa at different rates and durations. These variables are not directly measurable in the fossil record, but neoichnological observations and experiments provide informative analogues. Comparable to marine invertebrates from many phyla, a captive beetle larva burrowing over 2 weeks showed high rates of sediment disturbance within the first 100 h but slower rates afterwards. Tunnelling by earthworms and adult dung beetles is also inconstant—displacement of lithic material alternates with organic matter displacement, often driven by food availability with more locomotion when hungry. High rates of bioturbation, as with locomotion generally, result from internal and external drives, slowing down or stopping when needs are filled. Like other processes affecting sediment deposition and erosion, rates can drastically differ based on measured timescale, with short bursts of activity followed by hiatuses, concentrated in various seasons and ontogenetic stages for particular species. Assumptions of constant velocities within movement paths, left as traces afterward, may not apply in many cases. Arguments about energetic efficiency or optimal foraging based on ichnofossils have often overlooked these and related issues. Single bioturbation rates from short-term experiments in captivity may not be comparable to rates measured at an ecosystem level over a year or generalized across multiple time scales where conditions differ even for the same species. Neoichnological work, with an understanding of lifetime variabilities in bioturbation and their drivers, helps connect ichnology with behavioural biology and movement ecology.

**Supplementary Information:**

The online version contains supplementary material available at 10.1007/s00114-023-01833-0.

## Introduction and background

**Fig. 1:**
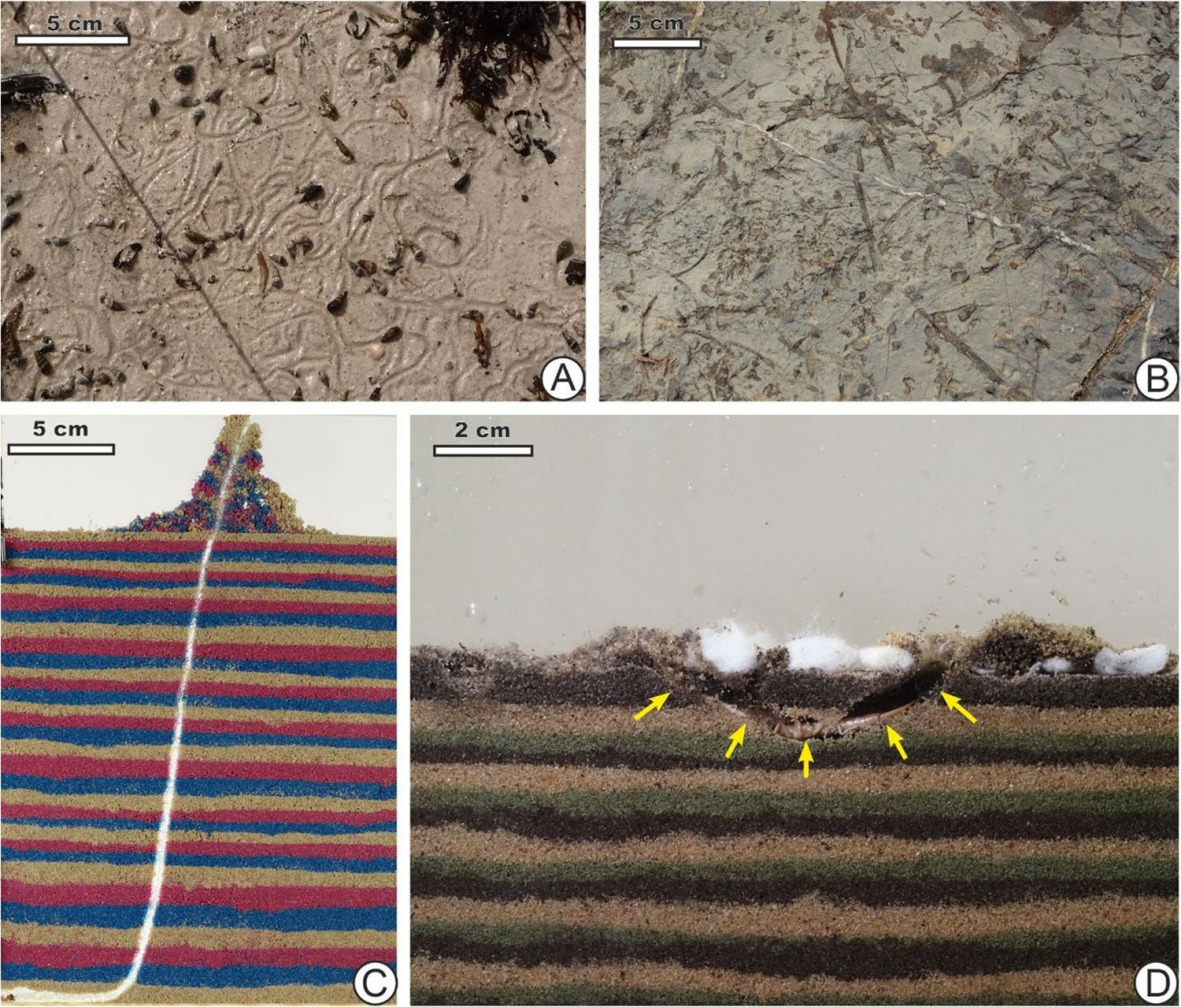
Though data on the amounts of bioturbation represented in a deposit are abundant in the geological record, estimates of speeds or rates remain elusive. **A** Snail (*Batillaria minima*) trails on modern marine carbonate intertidal mudflat, San Salvador Island, the Bahamas, produced within one tidal cycle, 12 hours (h). **B** Bedding plane from the Anisian limestones, High Tatric Unit, Tatra Mountains, Poland. Here, a horizontal bedding plane shows *Planolites* formed within the sediment on the sea floor, in a density similar to **A**, yet how much time was involved in their production is unclear. **C** Captive tiger beetle (*Cylindera arenaria viennensis*) larva dwelling burrow, produced in under 24 h, resembling the trace fossil *Skolithos*. **D** U-shaped shelter burrow from captive sand earwig (*Labidura riparia*), also produced within around 1 day, resembling the ichnofossil *Arenicolites*

Bioturbation, the transport and mixing of sediments and soils by organisms, is one of life’s great influences on the world’s marine and continental environments. Performed by macroscopic animals for over half a billion years, it continues at various rates across different places (Meysman et al. 2006; Wilkinson et al. 2009; Rogov et al. 2012; Tarhan 2018). Researchers of modern systems measure bioturbation rates, for instance, in mass or volume of material moved per year, and can study the timing of bioturbation on different scales with a variety of methods (e.g. Capowiez et al. 2001, 2011; Bastardie et al. 2005; Maire et al. 2008; Wilkinson et al. 2009; Solan et al. 2019). Researchers using ichnofossil records cannot easily make such estimates (as fine temporal resolutions are only rarely available; Allport 2022) yet have a wealth of data from palimpsests and snapshots of traces representing bioturbation from the deep past, as seen in horizontal bedding planes as well as vertical sections (Fig. 1). A highly disturbed surface produced by slow rates over a long time span, for instance, may not be easily distinguished from high rates over a short time span. To quantify amounts of bioturbation viewed from either vertical or horizontal surfaces, ichnologists have developed and used measures such as ichnofabric indices and other indices (Reineck 1963, 1967; Droser and Bottjer 1986, 1993; Bottjer and Droser 1991; Taylor and Goldring 1993; Miller and Smail 1997) that reflect the amount of modification to the original sediment.

Neoichnological approaches can bridge these approaches by examining bioturbation in real time while using ichnofabric indices or similar measures of areal coverage comparable to those used for fossil data. Though pauses, delays or gaps in the process of burrow-making and other forms of tracemaking would not be recorded, potential minimum times needed to create individual traces or trace assemblages may be estimated from living analogues, albeit with assumptions about similarities in tracemaker biology. For instance, Gingras et al. (2008) measured burrowing rates of marine invertebrates, including arthropods, bivalves and echinoderms, suggesting, for example, that the *Skolithos* ichnofacies may result from high population densities of animals across seasonal intervals and that the *Cruziana* ichnofacies could reflect moderate densities and short time spans.

A topic with implications for interpreting ichnofossils and their potential producers, which has rarely been discussed, is variability in the duration and rates of bioturbation even within a species or an individual’s lifetime. Changes in timing, duration and rates of locomotion are widely measured in biological research, for instance, in movement ecology (Nathan et al. 2008), an interdisciplinary area which Plotnick (2012) suggested could be integrated more strongly with ichnology. This paper examines several burrowing invertebrate animal species—a captive May beetle larva and dung beetles under experimental conditions in our study, as well as three earthworm species from an older study—in situations where inconstant disruption of sediment was observed on timescales ranging from minutes to weeks. Their bioturbation habits are compared with the previous literature. Possible causes of shared trends involving changes in bioturbation rates among many animals are discussed, as well as implications for interpreting ichnofossils.

The animals whose bioturbation was analysed in the study are all common and widespread terrestrial invertebrates in Europe. They come from two terrestrial groups within the Annelida and Arthropoda and thus provide a useful comparison to the various groups of marine invertebrates previously studied by ichnologists in terms of bioturbation trends that may be common in many animal phyla.

## Methods

### Beetle bioturbation observations

#### May beetle

Burrowing trends and bioturbation rates of the May beetle or cockchafer *Melolontha melolontha* were estimated from a wild-caught larval specimen in Poland in September 2020. It was housed in a custom-made 0.55-cm-thick glass-paned container, filled to an area of around 21 cm × 26.5 cm with alternating sand layers (3.1 to 8.6 mm thick) composed of blue-, pink- and yellow-coloured aquarium sand (*x̄*, mean = 0.57 mm, *σ*, standard deviation = 0.18 mm; the grain size was chosen to be similar to that of sand in dune habitats where the living species as well as some subfossil bioturbation traces had been found). It was photographed at points between September 11 and 25, 2020, starting after the larva was placed into the container and had already made a small initial burrow with the first observation (0 h) on September 11, at 12:06 pm. Measurements of the area disrupted cumulatively in a vertical section of sediment (comparable to the ichnofabric index) were taken based on the photographs and used to calculate bioturbated volume by multiplying by the container thickness. For each point, the total cumulative area of visibly bioturbated (i.e. noticeably transported or mixed, as observed from colour contrast) sand was calculated by digital tracing with CorelDRAW and area measurement with the software ImageJ. Filming of how the larva burrowed was also done, and the observations were briefly compared to previous studies.

#### Dung beetles

Adult dung beetles of the species *Anoplotrupes stercorosus*, wild-caught in October 2020, in the Niepołomice Forest, Sandomierz Basin, southeastern Poland, were placed in similar 1.5-cm-thick glass-paned containers filled with layered (2.9- to 15-mm layers) medium-grained sand (both artificially coloured and natural, collected from dunes in Poland in the area of the European Sand Belt; Uchman 2019; Łapcik et al. 2021; Hsieh et al. 2023; similar to those where the species has been observed in the field) with goat manure, forest mushrooms and other organic debris, placed as food on the surface. The manure formed a layer of 2–3 cm thick. Qualitative observations, including those from filming, of how the beetles bioturbated and moved sediment, as well as manure, were made. There were 16 beetles in different containers, with 3–4 per container, and the duration of filming was 3–4 h every couple of days when the food was placed.

### Earthworm bioturbation

Data and descriptions taken from an older study (Evans 1947) of three earthworm species kept in captivity, *Lumbricus rubellus*, *Lumbricus terrestris* and *Allolobophora* (*Aporrectodea*) *caliginosa*, were used to calculate how burrowed areas—either absolute area, where reference measurements were given, or relative area compared to the maximum burrow extent—changed over time. Evans (1947) illustrated, with diagrams of burrow areas shaded in black, how burrow systems progressed over periods spanning weeks. ImageJ was used to calculate areas and graph the total extent covered by burrows over Evans’ (1947) study period. Changes were then compared with descriptions of particular events in Evans’ study (e.g. food depletion).

## Results

### May beetle

**Fig. 2:**
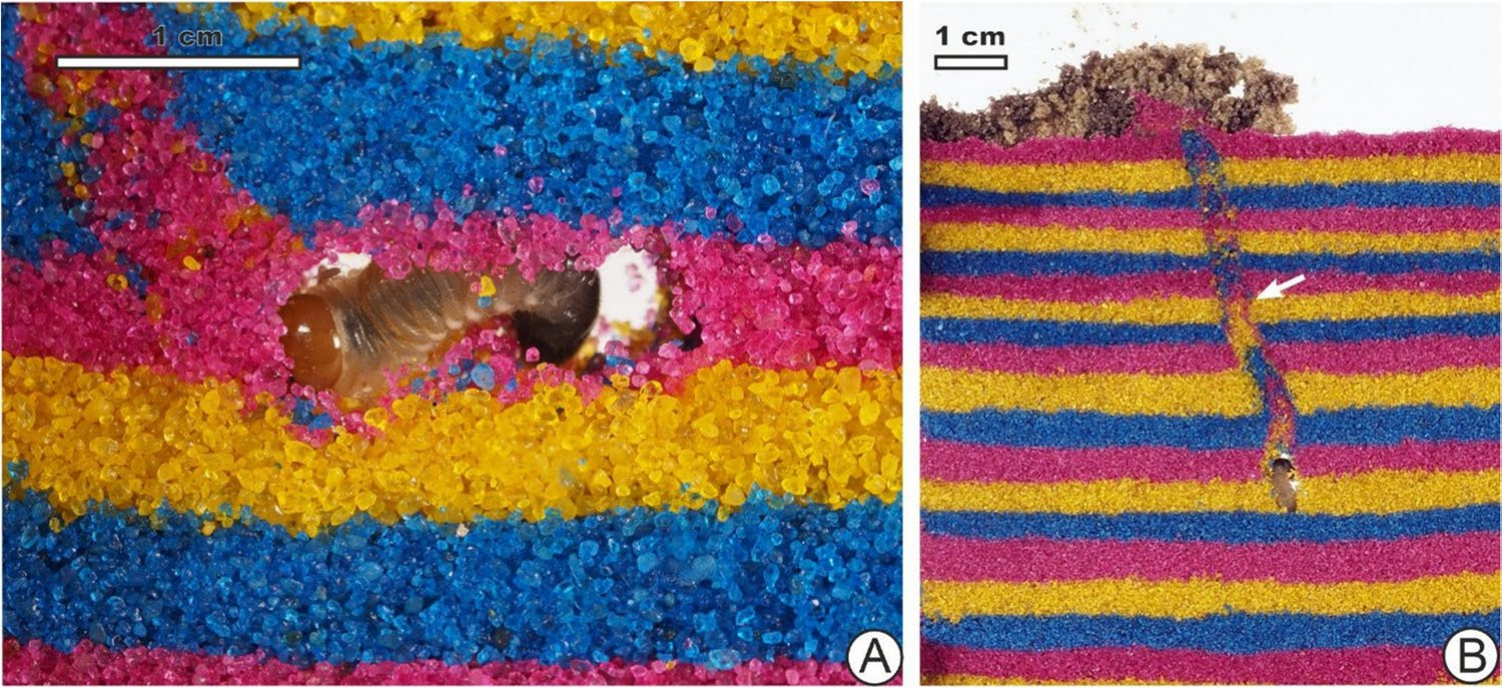
*M. melolontha* larva photographed during the process of burrowing. It is visible in its open chamber (**A**) through the glass panes. Sediment previously pushed and packed behind it is visible in the form of a meniscate burrow (**B**)

The larva of *Melolontha melolontha* created meniscate backfilled burrows in a manner consistent with that previously described by Schwerdtfeger (1939) and similarly observed by Counts and Hasiotis (2009) for masked chafer larvae (*Cyclocephala*), also in the Scarabaeidae family. *M. melolontha* backfills the space behind it with sand, leaving just a small open chamber for itself about as long as its own body (Fig. 2). It scrapes and shovels sand ahead of it with its head and mandibles, moving it towards its legs where it can be manipulated into a ball or packet, passed further along and then packed into the burrow behind it (see supplementary video, 00:07–01:16).

**Fig. 3:**
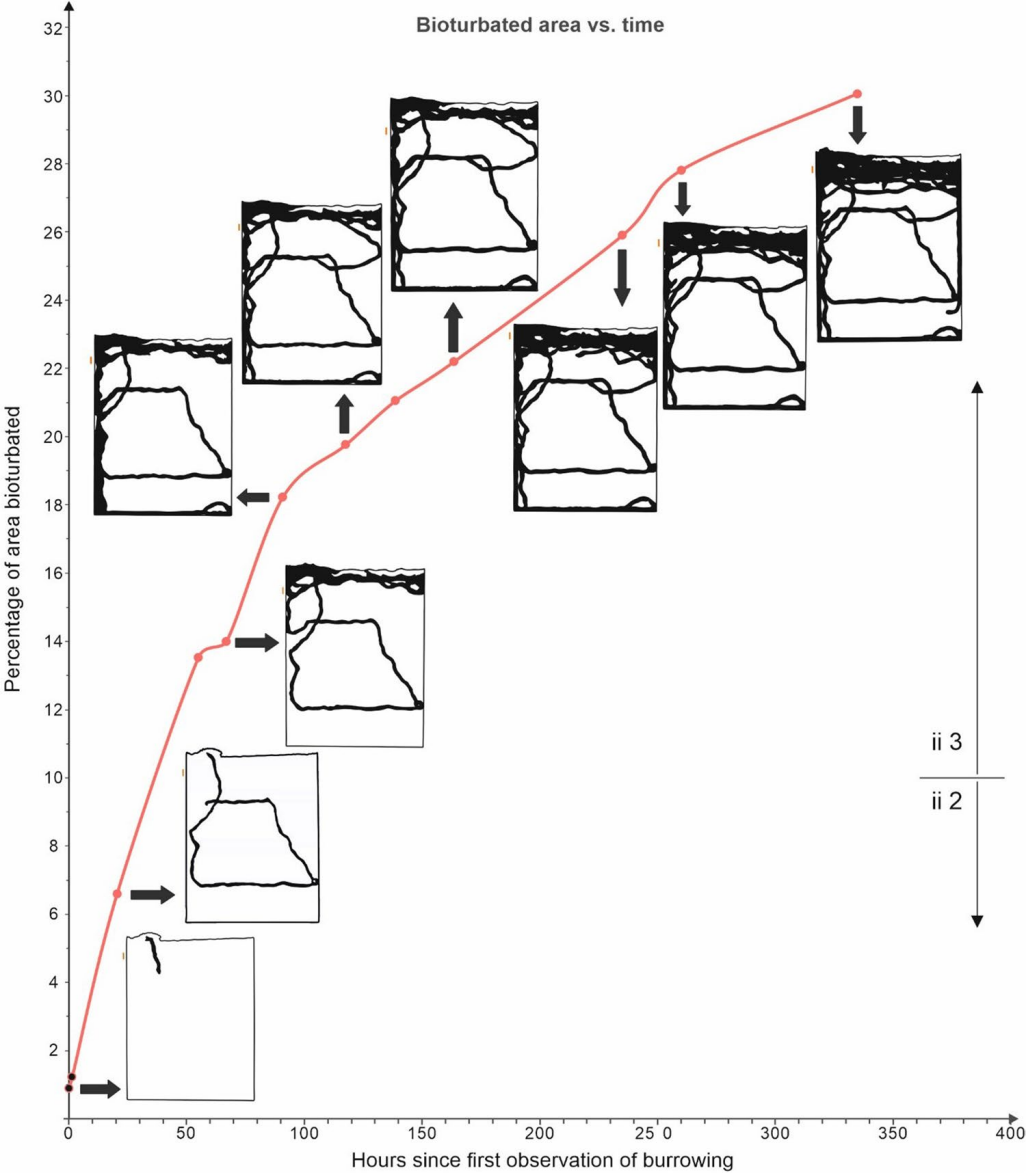
Cumulative bioturbated area over time measured from *M. melolontha* in the experiment as measured by percentage coverage and the ichnofabric index of Droser and Bottjer (1986). Diagrams show the bioturbated area in black in vertical view at selected time points

The bioturbated area as seen through the glass panels in vertical view (Fig. 3), which encompassed the backfilled burrow and mixed sand, was about 0.9% of the total area of sediment during the first initial observation (0 h) and, by 20 h, later reached 6.6%. At around 55 h, the area bioturbated was 13.6%, and slightly after 90 h, it was 18%, with the bioturbated extent changing noticeably slower later. For instance, it was 21% at 139 h, 26% by 235 h and finally 30% at a bit under 335 h, during the last observation. By the ichnofabric index (ii) of Droser and Bottjer (1986), a value of ii 2 would be observed on the first day, and on the second day, a value of ii 3 was reached, where it would remain until the rest of the experiment 2 weeks later.

**Fig. 4:**
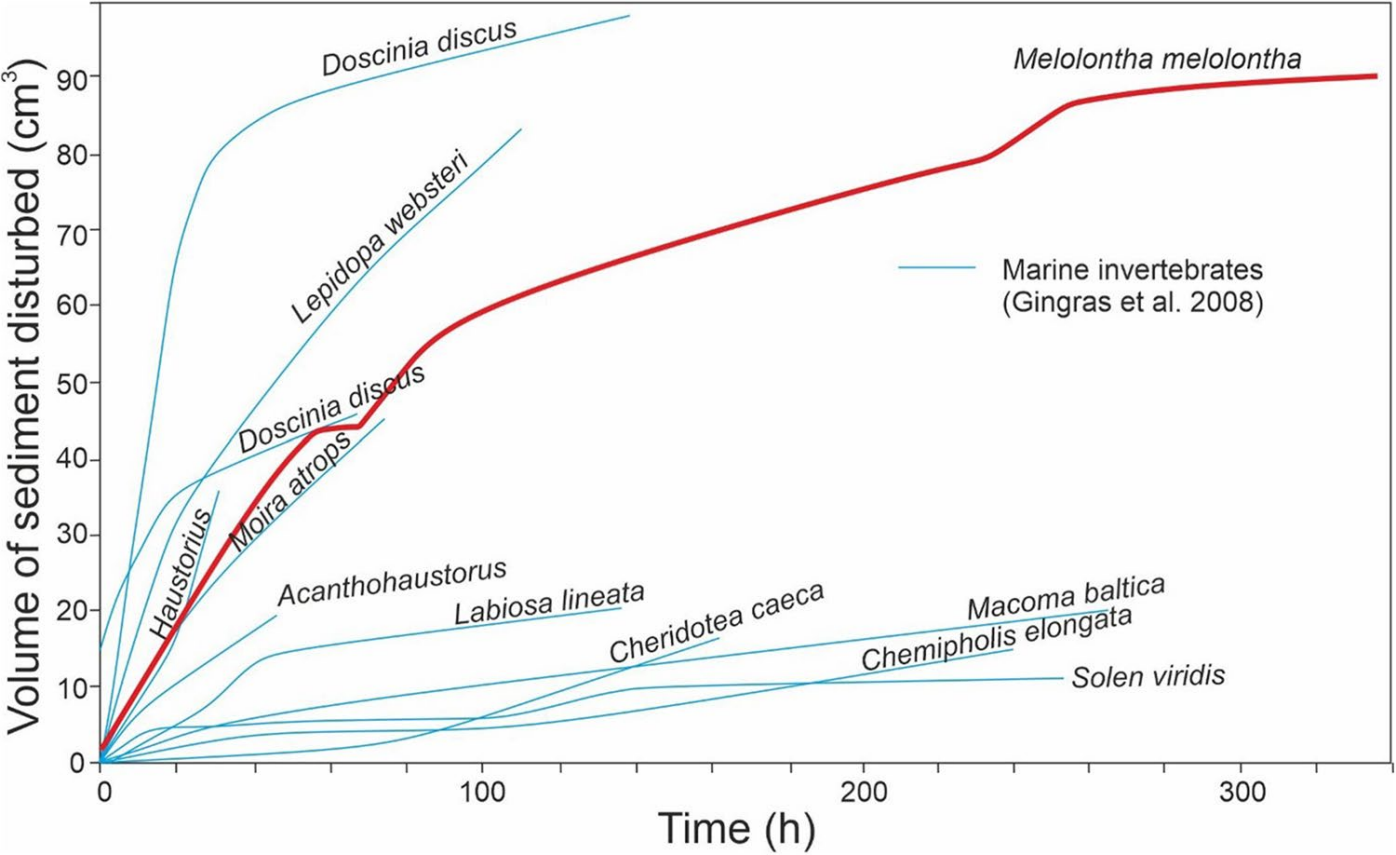
Volume of sediment disturbed by *M. melolontha* in the experiment over 300 h compared to several marine invertebrates studied by Gingras et al. (2008)

By the end of the experiment, slightly over 90 cm^3^ of sand was bioturbated. Calculated on average, bioturbation rate would be 0.26 cm^3^/h for the total duration, but as previously noted for other experiments (Gingras et al. 2008), it was higher initially and slowed afterward (Figs. 3 and 4)—rates for the beetle were 0.84–85 cm^3^/h for the first hour or first 20 h, around 0.69 cm^3^/h between 20 and 55 h, 0.21 cm^3^/h between 55 and 260 h and only around 0.05 cm^3^/h between 260 and 335 h. *M. melolontha* had bioturbation rates and trends similar to and within the range of many of the marine invertebrates studied by Gingras et al. (2008), including arthropods, bivalves and echinoderms, despite their taxonomic, anatomical, environmental and life habit differences.

### Dung beetles

**Fig. 5:**
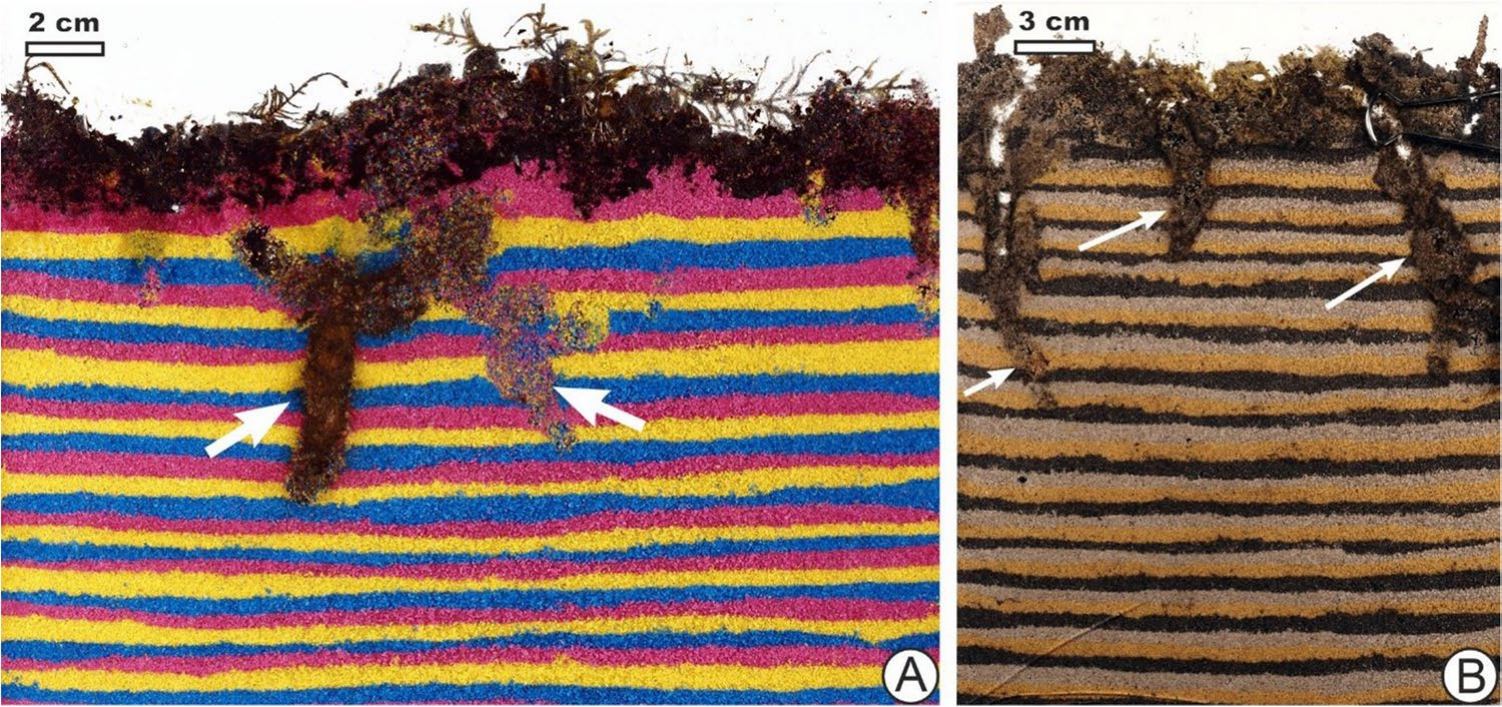
*Anoplotrupes stercorosus* burrows in captivity. **A** View of two large burrows or burrow branches visible near the centre of the container: one heavily filled with manure (centre left arrow) and one still relatively unfilled with manure (centre right arrow). **B** Burrows filled with a mixture of sand and manure

Multiple individuals of *Anoplotrupes stercorosus* placed in a shared container produced finger-like burrows 5–10 cm long in the sand underneath the manure and organic debris (Fig. 5, see supplementary video 05:07–07:23). Adults of these beetles dug by displacing sand around with their limbs, but did not ball up and move sand in discrete packages behind them (unlike the larvae of *M. melolontha* described above, others in the scarab family, as well as larvae of related earth-boring dung beetles in the group which they belong; Brussaard and Runia 1984; Counts and Hasiotis 2009) so their burrows filled with mixed sand generally lacked internal structure. Initial burrows in sand, possibly for sheltering, were created within a few hours of beetles being placed on the substrate. After such burrows were made, food in the form of manure or pieces of mushrooms brought from the surface was later observed to be packed into them by the beetles, until they were relatively full, which also took a few hours. The initial finger-like burrows were frequently modified into J or L shapes for food storage, and additional burrows of such shape could also be created and filled. Thus, bioturbation was intermittent and displacement of lithic material or sediment alternated with displacement of organic matter. High rates of movement of material were involved in creating the initial burrows and then filling them, but generally not other actions such as walking on the surface.

### Earthworm bioturbation

**Fig. 6:**
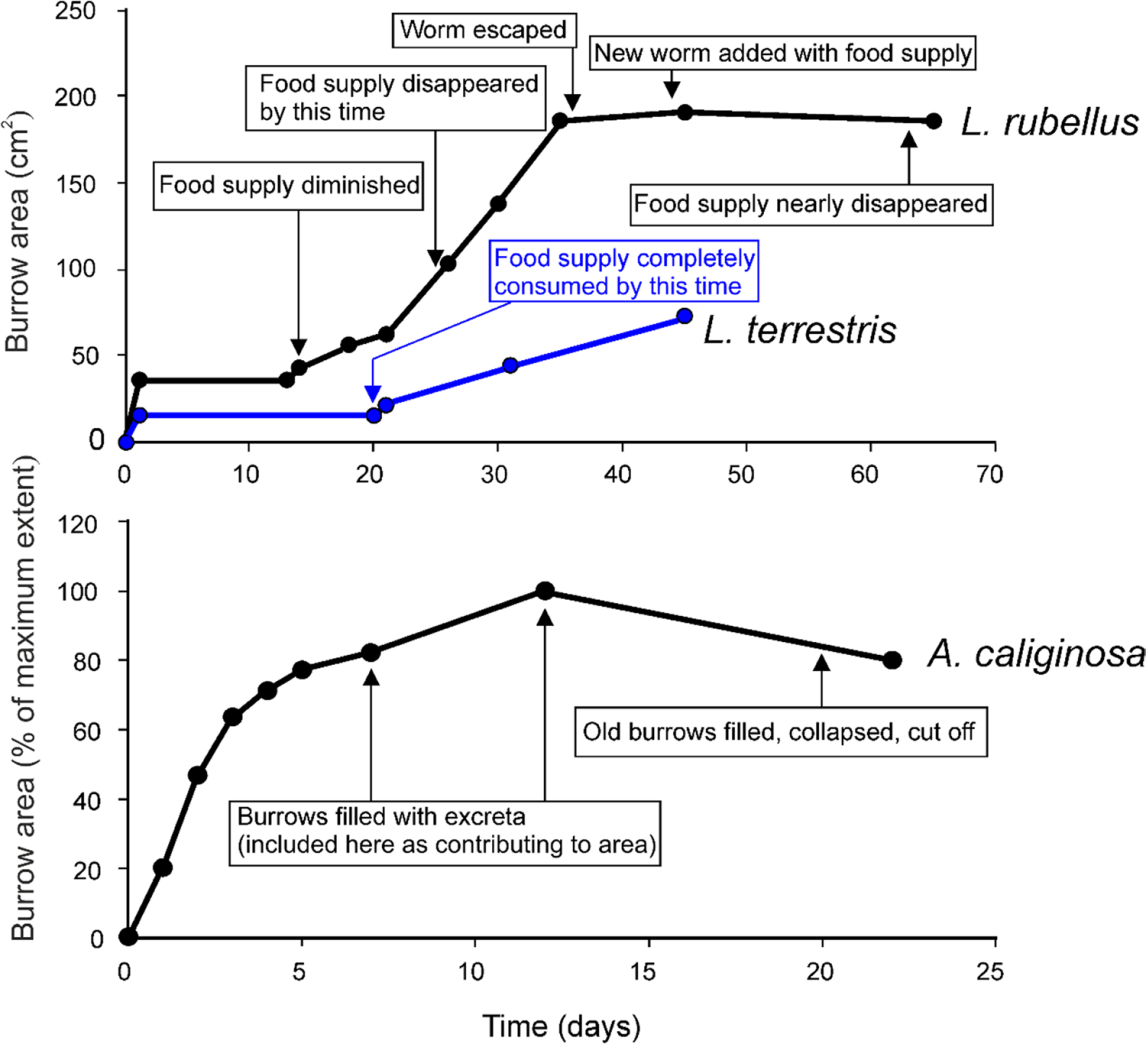
Change in burrow system area by three earthworm species, (top) *Lumbricus rubellus* and *Lumbricus terrestris* and (bottom) *Allolobophora* (*Aporrectodea*) *caliginosa*, through data taken from the study of Evans (1947). Descriptions of events relevant to or affecting the total burrow system area described by Evans are labelled on the plot

The three earthworm species studied by Evans (1947) all showed high rates of initial bioturbation as measured by burrow system area, either within the first day (Fig. 6, top graph) or first few days (Fig. 6, bottom graph), but would either have much slower rates of or major periods of no increase in measured burrow area afterwards. In particular, the *Lumbricus rubellus* and *L. terrestris* (Fig. 6, top) studied had no increase in burrow construction for 10–20 days after burrows were made on the first day. When food supply was diminished or disappeared, around the middle to end of the first month of observation, however, they resumed burrow-making at comparatively high rates once more for another span of time. As diagrammed and described by Evans (1947), burrow areas could be later lost to collapse and fill (Fig. 6, bottom).

## Discussion

### Comparisons across taxa

#### Similar initially high bioturbation measured in captivity or bare substrates

Most, if not all, of the captive animals described or reviewed above showed higher initial rates and then slowing rates of sedimentary disturbance (Figs. 4 and 6). Differences exist when comparing measured changes in burrow area (as with the earthworms), which decrease from collapse or fill later on, versus cumulative area of disturbed sediment (as with the May beetle), which cannot decrease, but nonetheless, the trend was similar. In ichnological research, the amount of cumulative change to a primary fabric at a given point is considered; even if burrows fill in or are compacted so that their volume or area is lowered, there is no true decrease as undisturbed primary fabric is not restored.

High bioturbation or burrowing rates when first introduced into bare substrates likely reflect animals quickly settling into their new environment, which can involve sheltering below-ground to avoid being exposed. Burrow construction occurred within an hour in the case of the May beetle larva, with initial scratching or disturbing of bare surfaces taking only seconds to minutes. For the May beetle in this study, earthworms from Evans (1947) and marine invertebrates by Gingras et al. (2008), sediment disturbance or burrow area change was often several times higher on the first day or first few days than later in the experiments lasting for weeks to months. Similarly, elevated bioturbation rates during burrow construction of the sandprawn *Callianassa kraussi* were noted by Pillay et al. (2007), who considered that this affected their shorter-term 2-week experiments but not their 2-month field experiments. Rützler (1975), researching bioeroding sponges, particularly *Cliona*, described how high initial penetration rates reflected mechanical stimulation and lack of competition, but that the rate curve flattens after 6 months, with further reduction from space or food limitation and increased competition. Gingras et al. (2008) discussed, for their marine invertebrate study, a distinction between what they called “intrusion time”, the time needed for an animal to initially move into the sediment and achieve the position or tier it would inhabit normally, and “subsequent time” with slower, steadier rates, when it is settled.

#### Slow rates, steady states?

**Fig. 7:**
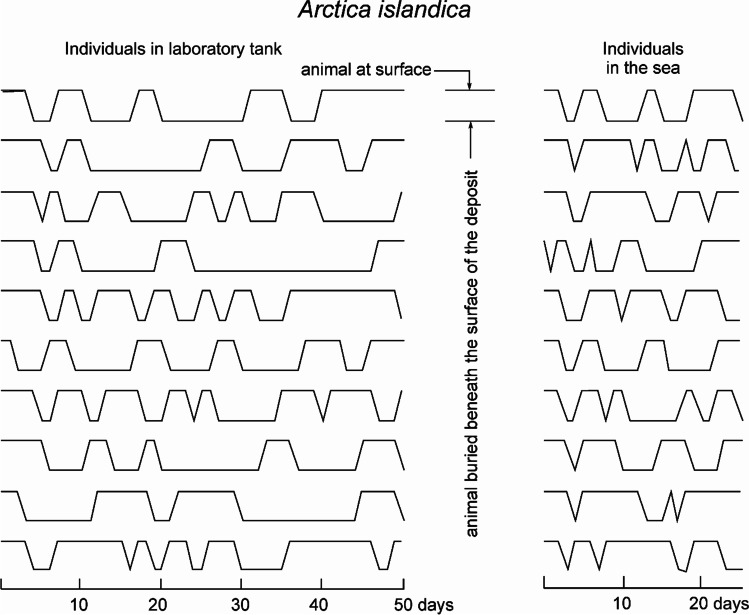
Intermittent burrowing activity by the bivalve *Arctica islandica* redrawn from part of Taylor (1976), where animals in the laboratory (left) and in the sea (right) all showed alternating periods of being at the surface and buried below it. Taylor (1976) suggested that the combined effects of energy-saving and predation avoidance may be involved, but that more research may be needed to explain and determine the prevalence of such patterns

Slower rates later might represent what might be called a steady state (e.g. Gingras et al. 2008). Steady-state assumptions or situations may need to be further examined, however. For instance, they could apply to organisms encountering more constant environments or predictable resource supplies across life, in contrast to those experiencing more changeable conditions. Rützler (1975) suggested that organisms making excavations to live, including many invertebrates but also non-animals such as algae, bacteria, lichens and fungi, heavily vary their activity based on substrate, environment and biotic interactions, while borers or scrapers such as mollusks, echinoids and reef fish erode calcareous material from constant activity needed for feeding. Observations from dung beetles and data from earthworms by Evans (1947) showed that even though the amount of bioturbation plateaued for a while past the first few days in captivity, after even more time, bioturbation rates became high again as animals burrowed in new places. Other research studies on earthworms, using methods that allow visualization within soil, such as X-ray tomography or radio-labelling (Capowiez et al. 2001, 2011; Bastardie et al. 2005), have resulted in observation of their burrow systems and movement trajectories in ways that can reveal when they are digging, travelling inside burrows or inactive, and thus, what bouts of inconstant movement might represent. For example, Capowiez et al. (2001) found that the beginning of their experiment had many burrowing phases while the end of the experiment had many displacement phases that show the earthworm to be reusing previously dug burrows. Taylor (1976) found the bivalve *Arctica islandica* burrowed intermittently while in captivity, alternating between being buried centimetres below the sand for periods of 1–7 days and being at the surface, without much rhythmicity and with individual variability (Fig. 7). Xie et al. (2018) described burrow construction in the polychaete *Nereis virens* as having two major phases with digging and consolidating of galleries taking 5–7 days, followed by maintenance where intermittent burrowing was done in existing burrows.

#### The influence of captivity

Many bioturbation experiments are performed with limited space, in captivity. In contrast to more sedentary species (e.g. suspension feeders with predictable food supplies and space requirements) or those with simpler habits, animals with complex behaviours such as active searching may turn back at boundaries and give up exploring more space after a time (potentially driving the spatial distribution of burrows in Fig. 3). It may be difficult to tell how applicable many results obtained from captivity may be to nature, especially if very motile animals have even simple kinds of learning or spatial memory (a topic that Plotnick (2012) described as potentially underexplored with regard to ichnology). For practical reasons, the experimental beetles, like Gingras et al.’s (2008) invertebrates, were studied in thin-walled containers which potentially may have changed their burrowing habits in comparison to those in nature. Nonetheless, some of the previously cited studies performed in more naturalistic settings, i.e. in the field or in wider tanks (e.g. those of Pillay et al. 2007), also have noted trends of higher initial rates of bioturbation in newly colonized substrates. This suggests that such trends may not be unusual in nature more broadly.

### The commonness of intermittent animal locomotion and its link to bioturbation

Sediment-disturbing locomotion can be considered a subset of locomotion in general, where intermittency has been noted to be common by biologists (Kramer and McLaughlin 2001; Bartumeus and Levin 2008; Bazazi et al. 2012; Paoletti and Mahadevan 2014). Kramer and McLaughlin’s (2001) survey found that animals exhibiting intermittent locomotion paused nearly 50% of their locomotion time, considering that, in contrast to previous steady-state assumptions of constant speed or the treatment of variable speed as unimportant in research, these breaks are too widespread to be ignored. Intermittent locomotion is described in these contexts as a stop-and-go movement within an activity (e.g. food search, seeking a mate, escaping danger or travelling from one place to another), rather than between types of activity (e.g. going from searching for to pursuing prey). Kramer and McLaughlin (2001), who focused on pauses from milliseconds to minutes, noted that many studies of locomotion lack the precision to record pause durations lasting under a second and that others ignore longer ones. They describe reasons for intermittent locomotion being common, including alleviating fatigue, stopping to pick up information and avoiding detection by predators, which would apply to underground burrowers as well as other bioturbators. Bioturbation variability across these and other, particularly longer, time scales is also worth noting, from an ichnological perspective.

### Reasons for discontinuity of measured bioturbation

On shorter time scales and smaller spatial scales, bioturbation is discontinuous due to factors related to the immediate environment and reactions to internal and external stimuli by an animal across its daily routine and through its lifetime. Many of these relate to Kramer and McLaughlin’s (2001) previously mentioned reasons for pausing from a biological perspective, but there are added factors when one takes a sedimentological perspective on how the deformation of the sediment actually relates to or records movement as opposed to pausing (discussed later). On broader temporal and spatial scales above the level of individual animals or single-species populations, shifts in community composition and major ecological and evolutionary changes become relevant (Table 1). Below, reasons for shorter-term discontinuous bioturbation for individual animals are discussed.

**Table 1:** Some examples of drivers of, or factors influencing, changing rates or discontinuities in bioturbation on a variety of temporal scales. Many of these factors also affect each other across multiple scales

Temporal scale	Examples
Seconds or less, to minutes and hours	Regular, repeated motions (e.g. digging strokes, discrete gaits, peristaltic contractions; Seilacher 1961; Stanley 1969; Savazzi et al. 1982; Savazzi 1985, 1991; Lin et al. 2019), immediate, quick responses to stimuli (e.g. gathering information, fleeing or stopping due to sudden danger or disturbance, hitting an obstacle); Kramer and McLaughlin (2001)
Hours, days, weeks, months	Circadian rhythms (sleep–wake cycles) (e.g. Atkinson and 1973; Riccio and Goldman 2000), responses to short-term weather (e.g. moisture changes with percolating rainwater), depletion of local food resources (e.g. Evans 1947)
Seasons, years	Seasonal movements (e.g. aestivation, hibernation, migration; Svendsen 1976; Hembree 2010), variability in seasonal resource availability for foragers and related energetics (e.g. Romanach et al. 2005), ontogenetic shifts (e.g. subterranean larvae to pupae to surface-dwelling adult), seasonal population changes (Dashtgard et al. 2014)
Years, decades, centuries, millennia	Changes in tracemaker densities across generations, colonization of new substrates by new populations of tracemakers, succession, habitat changes (e.g. Valdemarsen et al. 2018)
Evolutionary timescales (e.g. to millions of years or more)	Macroecological and macroevolutionary changes (e.g. evolution of new behaviours, tracemaker origination and extinction, co-evolution and escalation), very long-term habitat or biome shifts (e.g. change in climate, sea level or biogeography) (e.g. Herringshaw and Davies 2008; Tarhan 2018)

#### Locomotor constraints and gaits

Movement patterns in animals can often be deconstructed into individual small steps, such as a walking step or tail beat (Allen et al. 2018), and this also applies to digging. Step-by-step discontinuous movement is part of the natural burrowing gait in many animals, similar to surface locomotion such as walking where one body part (e.g. a foot) is fixed in place while another freely moves. Marine invertebrates belonging to diverse and disparate phyla, such as arthropods, brachiopods, echinoderms and mollusks, have terraces, ridges, ribbing or similarly described forms of sculpture on their shells or bodies serving as burrowing aid, allowing one part of their body to move forward, become anchored to prevent slippage, and then pull the rest of the body forward (Seilacher 1961; Stanley 1969; Savazzi et al. 1982; Savazzi 1985, 1991). Animals which dig with appendages, such as moles (Lin et al. 2019), also move through discrete identifiable strokes, so that closely spaced claw impressions on burrow walls may reflect points where the individual stopped and then pushed off once more (Gobetz 2005). Animals may pick up sediment in individual packets, pellets or balls; move them from place to place; or pack them into backfilled burrows behind them (as seen with this study’s May beetle). Thus, frequent stopping and starting of movements, often within the span of seconds to minutes, is to be expected based on physical mechanisms and constraints seen in diverse burrowers, even before considering larger-scale patterns of goal-oriented locomotion.

#### Fatigue

Intermittent burrowing also reflects energetic constraints, as moving through dense, cohesive solid material is incredibly costly compared to moving more freely through fluidic media such as when running, flying or swimming (Wu et al. 2015). Additionally, burrowers also often face low oxygen and high carbon dioxide levels deep in their burrows (Maclean 1981). Seymour (1973) measured rates of oxygen consumption by spadefoot toads (*Scaphiopus hammondii*) as they burrowed in soil. Digging was found to be intermittent at all tested temperatures, with calculations revealing that slight oxygen debts could be repaid during the periods of rest between bouts of burrowing; the author suggested that the toads could work more economically this way and avoid a large oxygen debt compared to if they dug continuously. Xiao et al. (2019) performed a thin-section analysis of the Ediacaran ichnospecies *Yichnus levis* associated with microbial mats, with observations suggesting that its tracemaker repeatedly moved in and out of the mat. They proposed that intermittent burrowing reflected the animal exploring an oxygen oasis where it could mine the mat for O_2_, against a backdrop of challenging redox conditions.

#### Bioturbation and fulfilling needs

Digging behaviour, like other movements generally (Nathan et al. 2008), is initiated by external or internal drives, e.g. for food, water or shelter, to escape from danger or environmental stress or to reproduce. Bioturbation stops when these needs are fulfilled, such as if the animal is satiated after feeding, has finished nest-building or successfully escaped, only to begin again if another need arises. The frequency of re-burrowing thus varies tremendously.

Sediment-ingesting animals feeding and burrowing at the same time, such as marine callianassid shrimp or terrestrial earthworms, might be expected in natural settings to have a relatively steady, continuous, excavation behaviour over much of their life. Callianassids happen to be well-known to ichnologists as long-proposed tracemakers for the common ichnotaxa *Ophiomorpha* and *Thalassinoides* (Bromley and Frey 1974; Netto et al. 2017). Stamhuis et al. (1996) studied the behaviour of captive *Callianassa subterranea*, making observations in periods of 1 h or 2 h randomly distributed over the 24-h day, and found that slightly more than a quarter of the overall time budget, which was a little more than 40% of the time that it was active and not resting, *C. subterranea* was engaged in behaviours categorized as burrowing, which involved sediment interaction and transport (the authors also discussed the assumption that behaviour during this time encompassed feeding while this sediment was mined for food and processed). In contrast to sediment-eaters selecting small organic particles, many below-ground terrestrial fossorial herbivores as discussed by Andersen (1987), including nematodes, insects and rodents, seek large, nutritionally dense but scattered plant organs such as roots, bulbs and tubers (the May beetle in this study is an example, though it was not fed during the experiment due to the difficulty of doing so in the laboratory set-up). Andersen (1987) also noted that these animals seem more patchily distributed in space and potentially vary temporarily more in abundance than above-ground herbivores. It may be interesting to consider if this trend would often result in more temporally and spatially discontinuous bioturbation.

**Fig. 8:**
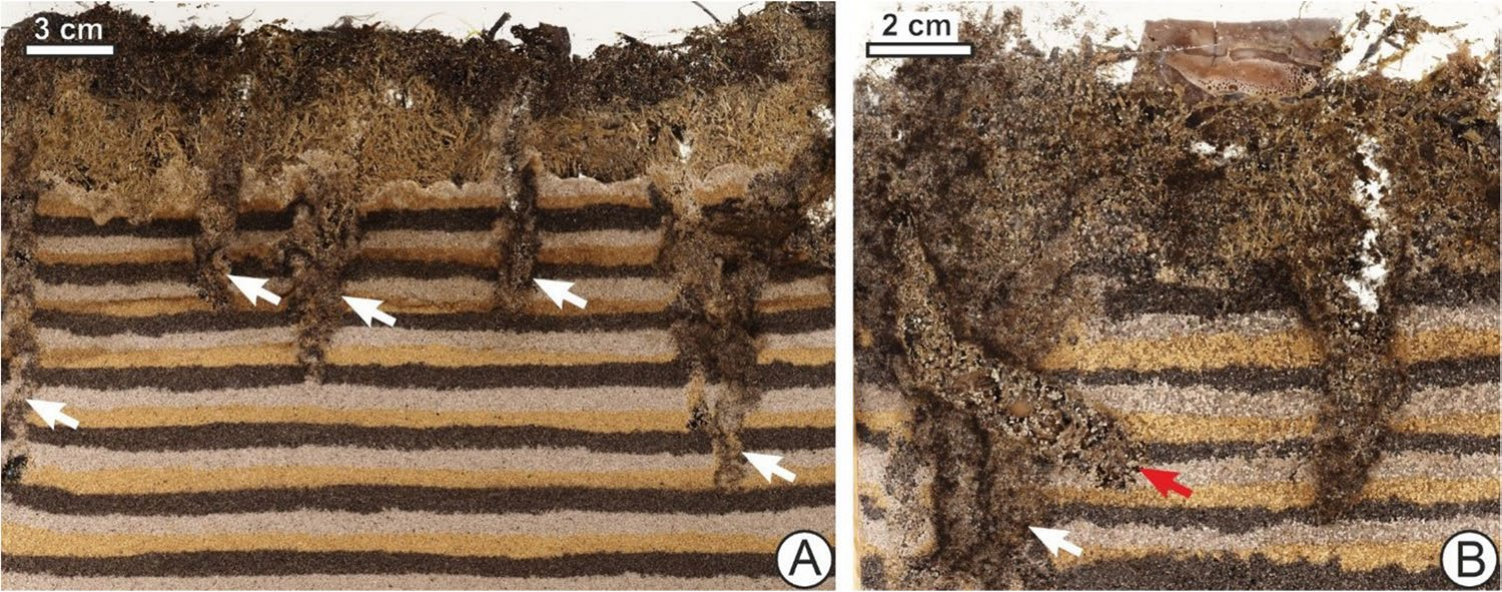
Bioturbation being influenced by food availability in *A. stercorosus* in captivity. **A** Initial burrows produced within a few hours after beetles were placed on the surface, indicated with white arrows. **B** J-shaped burrow (red arrow) that was later produced and filled with a hash derived from mushrooms placed on the surface as food. This burrow cross-cut or overprinted the previous burrows (white arrow)

Evans’ (1947) work on earthworms that showed burrowing to be triggered by depletion of food is consistent with research that other animals, including fiddler crabs (Genoni 1991) and mayfly larvae (Bachteram et al. 2005), also increased their burrowing or bioturbation when food supply was low. Consistent again with the idea that there are shared similarities in drives to bioturbate among marine and terrestrial animals alike, Gingras et al. (2008) described how a lack of food at the sediment–water interface for the scavenger *Chirodotea* made the arthropod use secondary behaviours to exploit secondary sources of food, but only after 2 days had passed. A trend in hunger driving more active or aggressive locomotion, more generally, has been previously found (e.g. Shen 2012). That food scarcity rather than abundance increased foraging movement had already been noted by Kitchell et al. (1978) and Young et al. (1985), who researched the deep seafloor and tied their findings to ichnological interpretation. If starvation is prolonged, however, there would be a decreased ability to bioturbate due to lack of energy, as Haider et al. (2020) studied in the bivalve *Mya arenaria*. Responses to increased or decreased bioturbation with hunger can be highly variable, so that generalizations are difficult without knowing the species, its lifestyle and the particular situation. Although many animals move or forage aggressively, others might attempt to wait out periods of low food by being inactive or dormant, and those which cache or hoard food, such as dung beetles, can draw on food reserves, for example in a burrow or chamber, whose creation relied on earlier bioturbation at a time when the individual was less hungry (Vander Wall 1990). For instance, new food supply in the form of forest mushrooms placed on the surface triggered the experimental *A. stercorosus* dung beetles to start moving and cutting the mushrooms, placing them below in new or already-made burrows, as if refilling their larders (Fig. 8).

Compared to burrows for feeding, burrows used for protection and shelter can be quickly made and used without having to be remade for a long time: for instance, Craighead and Craighead (1972), researching how grizzly bears in Yellowstone Park created dens for hibernation, found that major excavation was usually accomplished in 3–7 days, though with minor digging also occurring over a span of weeks; the duration of very strong pre-hibernation bioturbation by these bears would be quite short compared to their 5–6 months of winter sleep. A sand earwig *Labidura riparia* was found to produce a single U-shaped shelter burrow 1 day (Fig. 1D) after being transferred into a sand-filled container, but afterwards did not make any more burrows there for the remainder of 4 months, which was the majority of its captive life (Hsieh et al. 2022). However, some burrowing animals have dynamic, shifting, home ranges, such as the blind mole-rat *Spalax ehrenbergi* researched by Zuri and Terkel (1996) and silvery mole-rat *Heliophobius argenteocinereus* found by Šklíba et al. (2009) to, on average, excavate about 0.7 m of new tunnels a day, backfill around 64% of tunnels and make a new nest every month; some parts of the burrow system were treated as more permanent than others. Šklíba et al. (2009) suggested reasons for these habits, including access to concentrated food resources (like those described in Andersen 1987), avoidance of predators and parasites and ease of territorial defence. Šklíba et al. (2009) also contrasted the findings in these solitary mole-rat species, to the assumption by Nevo (1999) that home ranges become primarily fixed once they are established and used for a breeding season by all subterranean mammals, and also suggested that stability would be expected for social rodents with large burrow systems (Bennett and Faulkes 2000). Eldridge and Pickard (1994) found that funnel ants, *Aphaenogaster barbigula*, in semi-arid Australia had nest entrances that remained active for about 9 months, with their location changing on average twice a year; they noted that though soil removal seemed more marked after rainfall or in warmer months and seemed cyclic, there was no strong seasonal pattern or correlation with environmental variables.

A major difference between aquatic and terrestrial settings would be requirements to frequently maintain or remake burrows due to the increased mobility of aquatic sediments leading to easy collapse without continuous regular investment (Ruxton et al. 2014). This might theoretically lead to bioturbation being more regular or frequent among some aquatic tracemakers, particularly those that build semi-permanent or permanent dwellings, though it might also disincentivize others from engaging in much regular repeated costly burrowing in the first place. Ruxton et al. (2014) suggested that the physical difficulty of making complex nest structures that last multigenerationally plays a role in the extreme rarity of eusociality in the oceans.

Findings that bioturbation rates and durations vary widely among bioturbators based on their goals highlight the importance of accurately inferring the motivation behind their behaviours. For example, if burrows that were regularly producing temporary shelters were assumed to represent more permanent, infrequently built, long-term dwellings, the activity level of the tracemakers would be strongly underestimated. A deeper understanding of ethology can be crucial in correctly inferring amount, rate or duration of bioturbation.

#### Changing locomotion and substrates

Only a fraction of behaviours meaningfully modify substrates, displacing and deforming solid materials (Plotnick 2012; Vallon et al. 2016), so gaps between recorded behaviour need not reflect the absence of action in between. An animal whose bioturbation appears discontinuous, as read from a sedimentary record, may not be stopping and restarting but rather continuously moving, just entering or moving to new substrates without (or with low) potential for trace registration or trace preservation, before returning to the previous substrate once more.

Novack-Gottshall (2007) produced a detailed theoretical ecospace to quantify the life habits of organisms, and within his framework, substrate composition types were divided into biotic, lithic and fluidic, with the consideration that an organism can be situated above or within any of these substrate types. For instance, insects may fly through the air, a fluidic substrate; burrow through clay, a lithic one; or mine through plant leaves, a biotic substrate. By far, lithic substrates dominate in ichnological studies since fluidic substrates cannot preserve long-lasting traces, but biotic substrates are also researched (e.g. wood-borers). Many animals switch between these categories of substrates regularly. For example, though extremely uncommon in the record, flying animals may leave take-off and landing traces or disturb sediment while flying just above the sediment surface (Walter 1978; Vallon et al. 2016) while movements in between leave no trace. Similarly, fish may drag body parts while swimming along the bottoms of water bodies, in addition to feeding there in ways which leave traces which, again, are very rare (Martin et al. 2010). An animal may leave an uncompleted burrow in soil to walk on leaf litter on the surface before returning to finish it later. Measuring displacement of lithic material only (e.g. as is often done for comparisons with the fossil record since organic matter rarely preserves for a long term) might underestimate effortful transport of other kinds of materials as dung beetles do when filling burrows with food, and likewise, the effects of earthworms as they consume detritus or soil matter. For instance, in Fig. 6, parts of the burrow system filled with excreta by *A. caliginosa* were counted as contributing to burrow area in our neoichnological analysis, but what proportion of a burrow was likely occupied by such a material would not be easily known from fossil equivalents. Given that substrates are often heterogeneous within short distances in many spatial dimensions, these processes are extremely common, so movement paths may appear to be interrupted due to gaps in preservational potential. Small-scale heterogeneity within similar kinds of substrates (e.g. pebbles within a layer of sand or changes in moisture and compaction) may also regularly slow down or speed up bioturbation in ways that might not be easy to measure if not for direct observation from living tracemakers.

#### Changes with ontogeny and seasonal cycles

Many of the aforementioned processes drive bioturbation variability only within small fractions of a tracemaker’s life within the same life stage, but across ontogeny, there can be dramatic shifts in both the capacity or need to engage in burrowing and other forms of major sediment disruption. Some taxa strongly bioturbate only as mature adults, either because they are not massive or energetic enough or because they do not have any intimate ties with sediment when younger (e.g. benthic bivalves, polychaetes, echinoderms and other groups with a planktonic stage (Pedersen et al. 2008) that passively disperse are common in marine systems; Strathmann 1990; Burgess et al. 2016). For others, such as flying or surficial ground dwelling, actively dispersing insects with subterranean larvae, it may, in fact, be the earlier stages that perform bioturbating roles in ecosystems. Insects with complete metamorphosis in particular would have high variability across their life cycle and across seasons in substrate reworking: for instance, eggs laid in soil or other locations are non-motile and very newly hatched larvae may be too small to move much sediment, but become extremely strong burrowers as they grow. They may later settle in a spot for hibernation, aestivation or pupation, where their bioturbation ceases for a time until adult emergence, whereby adults could possibly maintain, increase or decrease their bioturbation potential relative to their younger selves. Adults often bioturbate in quite different ways than larvae: for example, the experimental adult dung beetles dug with limb movements, leading to areas of mixed, massive sand, and did not pack sand into meniscae in backfilled burrows as was seen in the May beetle and many other beetle larvae (e.g. Counts and Hasiotis 2009). Although these were not used in the study, adult May beetles, which are relatively short-lived compared to their larvae, also have different bioturbation habits as females lay eggs in burrows with vertical shafts and distinctive terminal chambers (Mikuś and Uchman 2013).

The bivalve *Panopea* burrows shallowly when young and lives deeply buried when older, having a foot that grows allometrically and becomes proportionally smaller until it is vestigial as an adult. This results in a loss of mobility over time with increasing size and weight, which can explain fossil assemblages of adults preserved in their burrows that likely succumbed to burial by storms (Łaska et al. 2019). Aspey and Blankenship (1976) studied burrowing in the gastropod *Aplysia brasiliana* (sea hare) and performed multivariate analysis on ten quantifiable parameters relating to it. Individuals fell into groups the authors labelled “efficient burrowers”, which were small, fast-burrowing and highly responsive to disturbance, and “inefficient burrowers”, which were large, slow and not very responsive, as well as a group that was intermediate but more similar to the former, seeming to be in a transitional state towards the latter. Aspey and Blankenship (1976) suggested that the “efficient burrowers”, which also appeared to actively engage in reproductive behaviours after emergence from burrowing, represented young and/or healthy individuals which might burrow in preparation for reproduction, while the “inefficient burrowers” represented the old and/or unhealthy, which burrowed to save energy due to deteriorating health or intolerance of environmental stress. Bioturbation across life stages may perhaps also appear to be more discontinuous than it actually is based on reading the sedimentary record, if bioturbation strength varies gradually across life, but there are some thresholds where it is strong enough, abundant enough or deep enough to be noticed as a signal in the record.

Especially in seasonal habitats (e.g. temperate or high-latitude regions, or places experiencing annually predictable flood or drought), animals may produce traces associated with nesting, egg-laying, brooding, pupation, hibernation, aestivation or emergence at very specific times of year. Many of these potential structures are interpreted in ichnoentomology research in soils and paleosols (Genise 2016). They may vary in durability or persistence before being destroyed or cross-cut by physical or biological processes afterwards (e.g. flooding, burrow collapse, reworking by life activities later in the season) that occur either predictably or stochastically, and also be palimpsested and time-averaged with one another. If tracemaking was disproportionately concentrated in some parts of the year and one was unaware of or lacked data on seasonality, one might be unable to distinguish high bioturbation rates concentrated within season(s), with low rates other times of the year, from a situation where a more moderate bioturbation rate was present throughout the year, based on ichnofabric alone. For instance, Romanach et al. (2005), studying seasonal rainfall-related effects, found that an increase in soil moisture initiates a burst of digging in the pocket gopher *Thomomys bottae* in California when soil is easily workable (but before plant growth has time to respond), with burrowing activity declining afterward and then reaching a steady rate which can be supported by vegetation growth. Romanach et al. (2005) also described how burrows collapse during the dry season and that the food and energy needed to repair them may not be available until the next rainy season.

Abundance of individuals across seasons or years in a locality or region can play a major role in bioturbation intensity. In the case of May beetles, previous research in parts of Europe on the species *Melolontha melolontha* and *Melolontha hippocastani*, both studied for their economic impact on agriculture and forestry, have shown multi-year cycles in abundance (Švestka 2010). Švestka’s (2010) work described regions heavily hit by swarming in particular years, monitored with light traps that caught the adults. Although it might be more difficult to directly survey larval abundances to link them to those of above-ground adults, people in farming communities where the beetles occur have long noted year-to-year variability in numbers of larvae observed below-ground, such as when harvesting potatoes (Uchman, pers. comm.).

### Implications for interpretation of trace fossils

#### Interpreting movement paths

As previously discussed, initial bioturbation (e.g. new burrow construction) on undisturbed or fresh substrates can be faster and stronger or leave more durable traces than bioturbation later on that might include modifying or maintaining the burrow or moving within it after it is complete. Measured amounts of bioturbation, using some indices, may also plateau after reaching an upper limit if there is no more untracked or available space (Wheatcroft et al. 1989), and subsequent movement reworks the same material again. In such situations, an accurate measure of total movement by organisms repeatedly in the same areas may not be practical from the extent or coverage of a trace; nonetheless, the area or volume of bioturbated sediment puts a constraint on all possible spatial positions previously occupied by them and the minimum amount of movement needed to produce the trace.

**Fig. 9:**
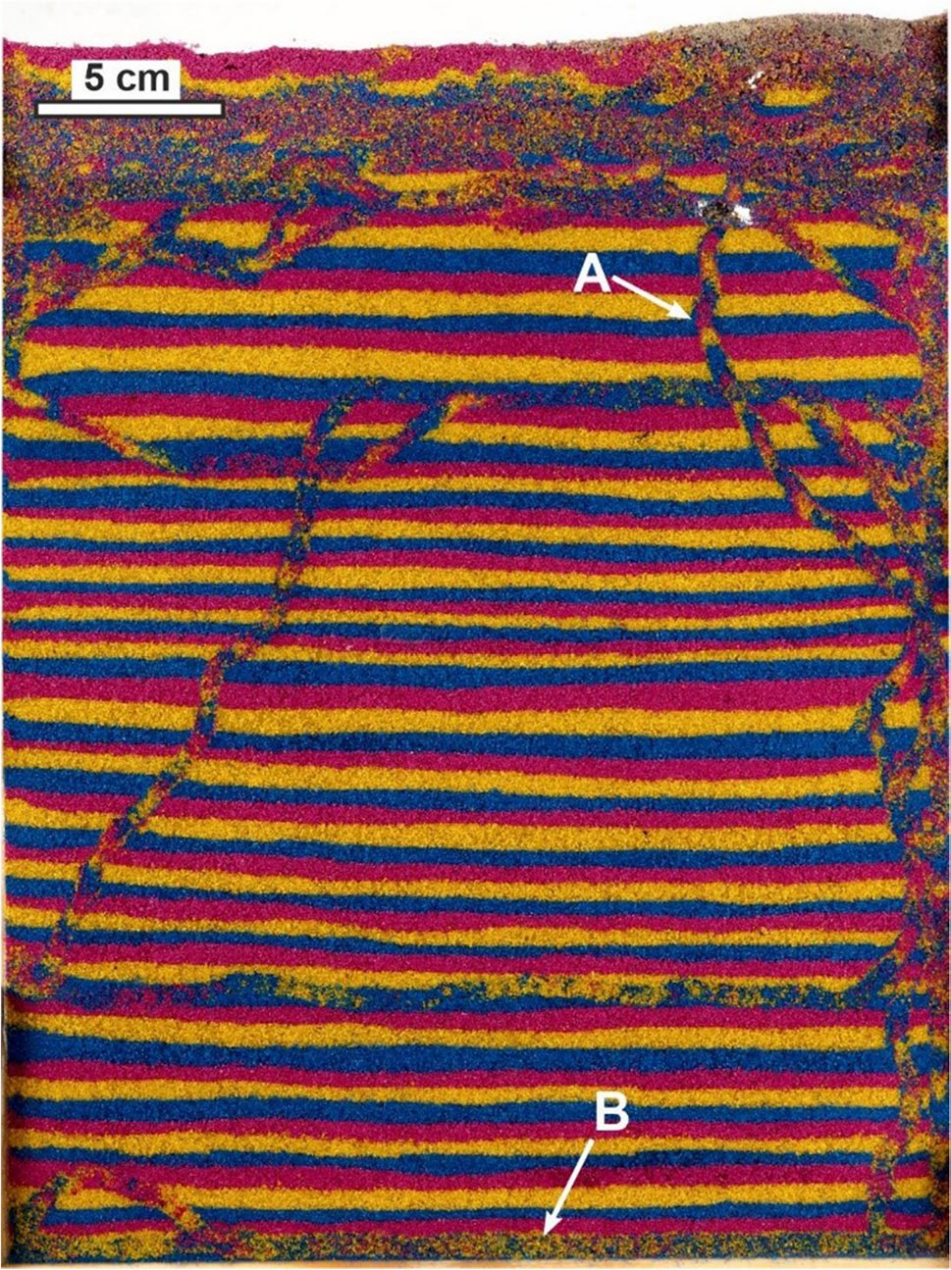
Backfilled menisci can indicate directions that animals, in this case the experimental May beetle larva, moved as they packed material behind them (**A**), but repeated back-and-forth movement (**B**) can erase this information, so that further motion along the same path is left unrecorded. Thus, it may be difficult to tell if plateauing of visibly bioturbated area reflects a true slowing down of bioturbation rate, or merely repeatedly bioturbating the same areas

**Fig. 10:**
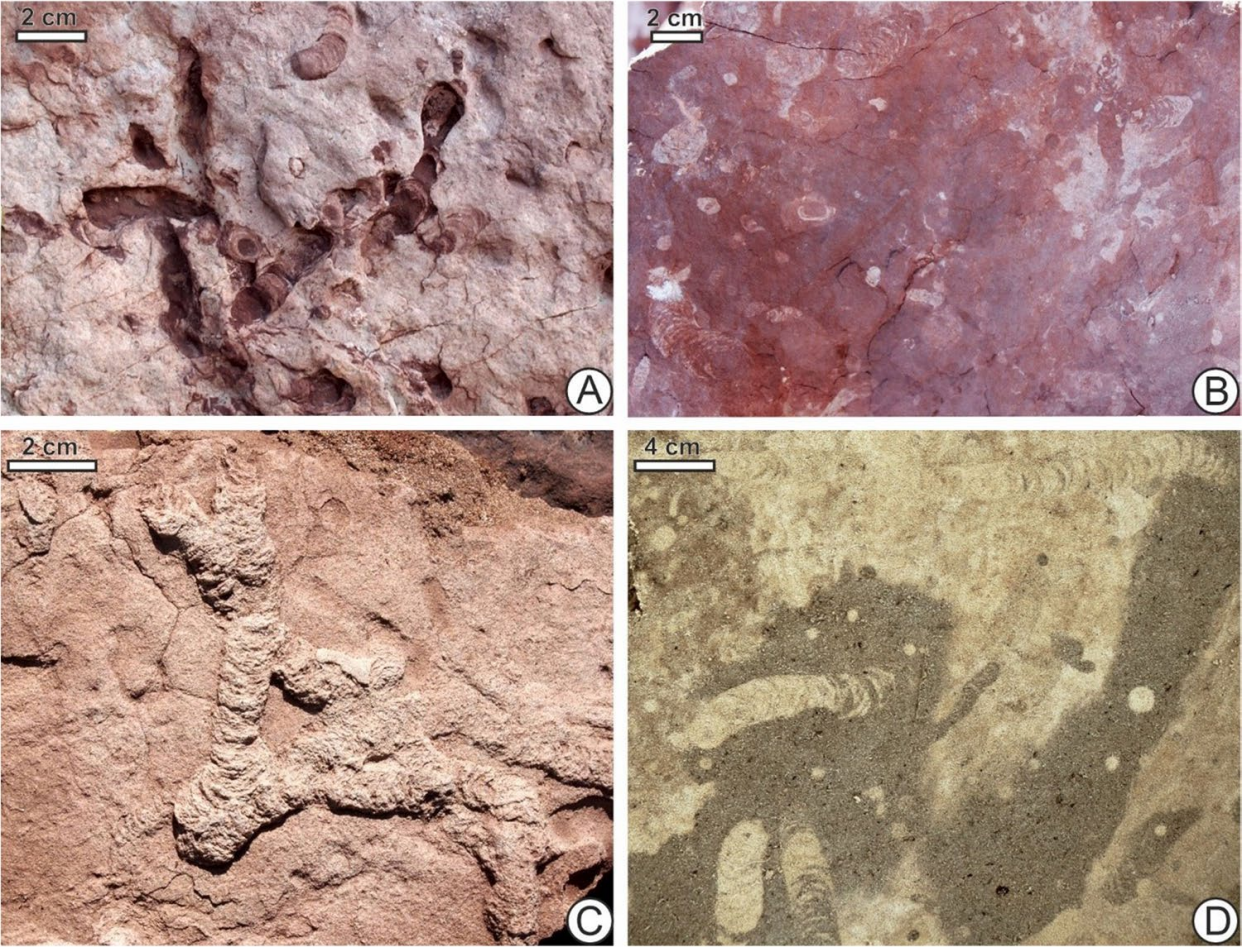
*Naktodemasis bowni* (or *Taenidium* isp., according to some opinions) on horizontal surfaces. **A**, **B** Middle Jurassic fluvial deposits, Tafaytour, Morocco. **C** Late Cretaceous fluvial deposits, Villa El Chocón, Rio Negro Province, Argentina. **D** Subsoil on Holocene dune, Wylewa, SE Poland

As with surface trackways where the orientation of footprints can be observed, subsurface traces sometimes indicate direction of movement, for instance in situations where backfilled menisci can be seen (Fig. 9A). Many previously described fossil burrows, such as *Naktodemasis* or *Taenidium* (Fig. 10), have these features. However, enough repeated travel across the same path can erase these signals (Fig. 9B). Even if one cannot predict if disturbed sediment was traversed only once, or multiple times and in which direction(s), undisturbed sediment at least can be used to suggest where movement was absent.

#### Knowledge of movement rates, optimal foraging and energetic efficiency

A biological topic with a long history of theorizing and study with trace fossils is optimal foraging (Richter 1924; Seilacher 1967; Kitchell 1979; Leighton 2002; Sims et al. 2014), particularly assessing the effectiveness of areal coverage from fossilized movement paths, then used to infer the efficiency of either searching or gathering food. Plotnick (2012) pointed out one of the limits of analysing movement with fossil data, which was the lack of absolute temporal information. Knowledge of absolute rates is needed to understand energetic efficiency in living systems today. For instance, Dorgan et al. (2011) calculated the energy required per distance travelled for the polychaete *Cirriformia moorei* while it extended burrows by fracture as well as displacing sediment. Dorgan et al. (2011) discussed that despite burrowing being indeed more costly per unit of distance than other forms of locomotion as previously suggested, in practice, worms have only very small metabolic increases between burrowing and resting due to locomotion being slow, so burrowing was inexpensive per unit of time. As previously discussed, burrowing animals may often frequently rest between digging, which can be more economical than digging continuously (Seymour 1973). Depending on a species’ lifestyle or environment, frequent acceleration, deceleration and changing speeds or gaits for locomotion in general might have different energetics than steady locomotion (Blickhan and Full 1987; Full and Weinstein 1992; Kramer and McLaughlin 2001; Minetti et al. 2013; Nauwelaerts et al. 2015). Thus, actually understanding the energetics involved for a fossil tracemaker is difficult without knowing the absolute and relative time required to complete steps of a movement path or a burrow.

Plotnick (2012) discussed how studies of fossil paths often assume continuous locomotion (“In most cases, it is necessary to assume that velocity within a path was constant”, p. 466). Yet discontinuous locomotion, including when tracemakers rest or pause for periods of time at points along their movement path before continuing again, triggered by different stimuli at different times, is common enough that this assumption can easily be violated (Kramer and McLaughlin 2001). For example, Ekdale and Bromley (2001) described a compound trace fossil interpreted as being made by a burrowing cleft-foot clam that paused five times to deposit-feed and then moved on to a new location. They considered that this compound trace could represent a few hours or no more than a day and a night, in the life of its maker. Pausing can but does not always leave traces which may be identified as ‘cubichnia’ (Vallon et al. 2016). Falkingham and Horner (2016) recorded experimental traces of terrestrial movement by lungfish, comparing them to the trackways of early tetrapods. They found that while the lungfish generally did not show continuous locomotion and paused for seconds to tens of minutes, tracks made during continuous motion were indistinct from those made during stop-start locomotion. They mentioned that this was comparable to previous observations from birds showing that stopping mid-stance had no recognizable effects on surface track morphology. It may be theoretically useful to average together periods of non-locomotion with fast locomotion, but information about when it would be appropriate to do so, and the relative duration of each may be lacking. Additionally, backtracking or reversal, and repeated movement along the same path, would also affect energetics, but may not always be recognized in ichnofossils, particularly burrows (Figs. 8 and 9), and might be undercounted in terms of amount of estimated movement.

Nonetheless, there is much potential for research with modern systems to help in these types of question. An example is the study by Miguez-Salas et al. (2022) of deep-sea echinoid trails and their relation to nutrient distribution, one of the few to incorporate detailed information, with time lapse camera, about speed and time durations between steps in a living analogue, into the interpretation of ichnofossils. In the study, they described straight movement patterns characterized by high-speed periods of echinoids covering large distances quickly (> 100 mm/h), followed by long periods of slow, sinuous movement (< 100 mm/h) with many turns and cross-cuts.

### Parallels between discontinuous bioturbation and other sedimentary processes

In a highly influential paper, Sadler (1981) discussed the extreme variability of measured sedimentation accumulation rates, where data from almost 25,000 rates spanned at least 11 orders of magnitude, and how this was driven by unsteady and discontinuous processes. A high sedimentation rate in a short time interval may be followed by a long span of little or no sedimentation, and Sadler (1981) described how longer intervals often incorporated more frequent and longer hiatuses so that there was a trend of falling mean rate with measured time span.

While bioturbation rates have not usually been systematically compared on such grand scales, we consider that the existence of discontinuities might also be similarly relevant. For example, an animal that produces a hibernation den (such as the bears studied by Craighead and Craighead 1972) may have a high bioturbation rate if measured only during a few days in autumn where the bulk of the structure is produced, but this rate would be much lower if averaged together with the following half of the year of inactivity or negligible bioturbation. This may be analogous to how high erosional or depositional rates are recorded during extreme events such as storms or landslides in places normally quiet most of the time in between. Cole (1995) reported fractal time variability in animal behaviour while examining activity patterns in *Drosophila* so that episodes of what appeared to be continuous activity had within them shorter episodes of inactivity in a self-similar way; flies appeared more inactive when the temporal scale of recording was finer. Kramer and McLaughlin (2001), discussing Cole’s (1995) work, considered that it was still not known to what extent this applied to intermittent locomotion. Similar types of intermittency having been discovered in sedimentary and behavioural processes provide a major insight for the study of bioturbation and ichnology, since the discipline lies at the intersection of both.

In a community of tracemaking animals, many animals are resting or pausing at a given time. Measured speed of the snail *Batillaria minima* from trail lengths representing either 15 min or 0.5 h of movement on a tidal mudflat was found to average 23.4 mm/h by Hsieh (2020), but the distribution of speeds was heavily right-skewed and nearly half the snails did not move during the interval. Thus, the average of non-resting (> 0 mm/h) speeds would be 42.9 mm/h, and by contrast, the maximum recorded speed among them was 134.4 mm/h. Subterranean or burrowing animals that spend most, if not all, of their life underground are of great interest ichnologically and have rarely been studied within the framework of movement ecology, with one example being research on the worm lizard *Trogonophis wiegmanni* by Martín et al. (2021); these authors also found a strong right-skewed distribution in its movement where most individuals covered small distances and areas per day, and that animals did not appear to move in a meaningful way on almost a third of the days.

Measured short-term bioturbation rates with experimental animals in lab settings may be high because they are studied precisely when they are most active or eager to excavate (e.g. when exposed on bare substrates and seeking shelter, when hungry and searching for food or when agitated). This may sometimes reflect values closer to their potential maximum rates, and thus be different from those examined by e.g. looking at naturalistic rates of soil mounding, burial rates and mixing in the field over the course of a year, where various periods of rest take place. Bioturbation studied on different time scales may be used for dissimilar applications in different disciplines: for example, those that study bioturbation within seconds, minutes or hours may be interested in observing locomotor mechanisms or neoichnological work involving tracemaker identity, while those studying rates within years, decades or longer may be interested in ecological succession, soil development and geomorphological change.

## Conclusions

Animal locomotion, including burrowing and other sediment-displacing actions, is changeable in speed/rate and mechanism and discontinuous across a number of time scales and within stages of an individual’s life; this has become increasingly recognized and incorporated in modern behavioural ecology and movement ecology (Kramer and McLaughlin 2001; Nathan et al. 2008), which can also inform ichnology (Plotnick 2012). This was also observed with the experimental May beetle larva showing high initial, and later slower, rates of measured bioturbation, consistent with previous data from captive marine invertebrates. Data from dung beetles and earthworms also show burrow areas can remain unchanged or little-changed for days to weeks before burrowing resumes once more for food-related reasons. For dung beetles and earthworms, movement of organic material can occur alongside or alternate with displacement of sediment, so that records of bioturbation over time on or within lithic substrates might appear discontinuous.

Thus, assumptions of constant speeds and steady states, previously used for simplicity, may not hold in many situations and across measured scales. Ultimately, this variability is driven by changes in both external (e.g. food availability, danger or physical stress on the surface) and internal (e.g. biological rhythms and ontogenetic changes) factors, which are hard to directly infer and model in fossil data. A diversity of modern analogues, which remain to be studied, can provide important insights from ichnofossil records that would otherwise be missing (e.g. Miguez-Salas et al.’s (2022) research on sea urchins complementing the rich history of research on optimal foraging on the seafloor from fossils, e.g. by Richter 1924, Seilacher 1967, Kitchell 1979, Sims et al. 2014 and others).

More cross-collaboration, not only between researchers on past and present systems but also those who work with bioturbation on different temporal and spatial scales, might be promising in furthering our understanding of how this important process shapes both the biosphere and the physical earth, and how we can interpret it in the past.

## Supplementary Information

Below is the link to the electronic supplementary material.

Supplementary file1 (DOCX 12 KB)

Supplementary file2 (XLSX 25 KB)
