# Supplementary material for: Intermittent and temporally variable bioturbation by some terrestrial invertebrates: implications for ichnology
Source: Naturwissenschaften. 2023 Mar 7;110(2):11. doi: 10.1007/s00114-023-01833-0 (PMC9992032; doi:10.1007/s00114-023-01833-0)
Supplement: Supplementary file 1 — Supplementary file1 (DOCX 12 KB) [file 114_2023_1833_MOESM1_ESM.docx]

Link to supplementary video

<https://data.mendeley.com/datasets/xn7p5f4c63/3>
